# Correction: First insights into coral recruit and juvenile abundances at remote Aldabra Atoll, Seychelles

**DOI:** 10.1371/journal.pone.0295829

**Published:** 2023-12-07

**Authors:** The PLOS ONE Staff

There is an error in Table 1. Several asterisks in Table 1 (in the two columns indicating percentage change) are wrong. Please see the correct Table 1 here. The publisher apologizes for the errors.

**Table 1:** Information on coral juvenile abundances and percentage hard coral cover at Aldabra between 2014 and 2019. Asterisks indicate significant differences between years; n.a. = not available.

Measure	Location	Water depth	Pre-bleaching^a^ 2014/15		Post-bleaching 2016		Post-bleaching 2019		% Change 2014/15–2016	% Change 2016–2019	% Recovery^b^
			Mean	SE	Mean	SE	Mean	SE			
Coral	West	Shallow	20.6	1.9	8.3	1.0	21.4	1.9	-60*	61*	104
Juveniles	West	Deep	14.3	5.1	7.7	0.8	22.9	1.8	-46	66*	160
(no. m^-2^)	East	Shallow	8.3	0.8	7.9	1.0	24.1	2.6	-6	67*	290
	East	Deep	11.5	2.7	5.5	1.0	16.6	1.9	-52*	67*	144
	Lagoon	Shallow	n.a.	n.a.	7.7	1.6	29.9	5.3	n.a	74*	n.a
Coral	West	Shallow	34.1	2.9	15.9	1.8	23.7	2.0	-53*	33*	69
cover	West	Deep	20.8	1.9	8.6	1.0	13.6	1.2	-59*	37	65
(%)^c^	East	Shallow	23.6	4.2	9.7	0.8	12.5	1.5	-59*	22*	53
	East	Deep	10.1	2.9	5.1	1.1	5.0	1.2	-49*	-0.02	55
	Lagoon	Shallow	32.3	5.0	19.6	4.5	30.0	4.7	-39	35*	93

^a^ 2014: Coral cover; 2015: Coral juveniles

^b^ Percentage of pre-bleaching value reached in 2019

^c^ Significant changes based on previous work conducted at Aldabra [34]; note, displayed here are mean coral cover values and the respective percentage changes and not back-transformed model estimates and the respective percentage changes shown in previous study [34].
