# Supplementary figures and images for: First insights into coral recruit and juvenile abundances at remote Aldabra Atoll, Seychelles
Source: PLoS One. 2021 Dec 7;16(12):e0260516. doi: 10.1371/journal.pone.0260516 (PMC8651144; doi:10.1371/journal.pone.0260516)

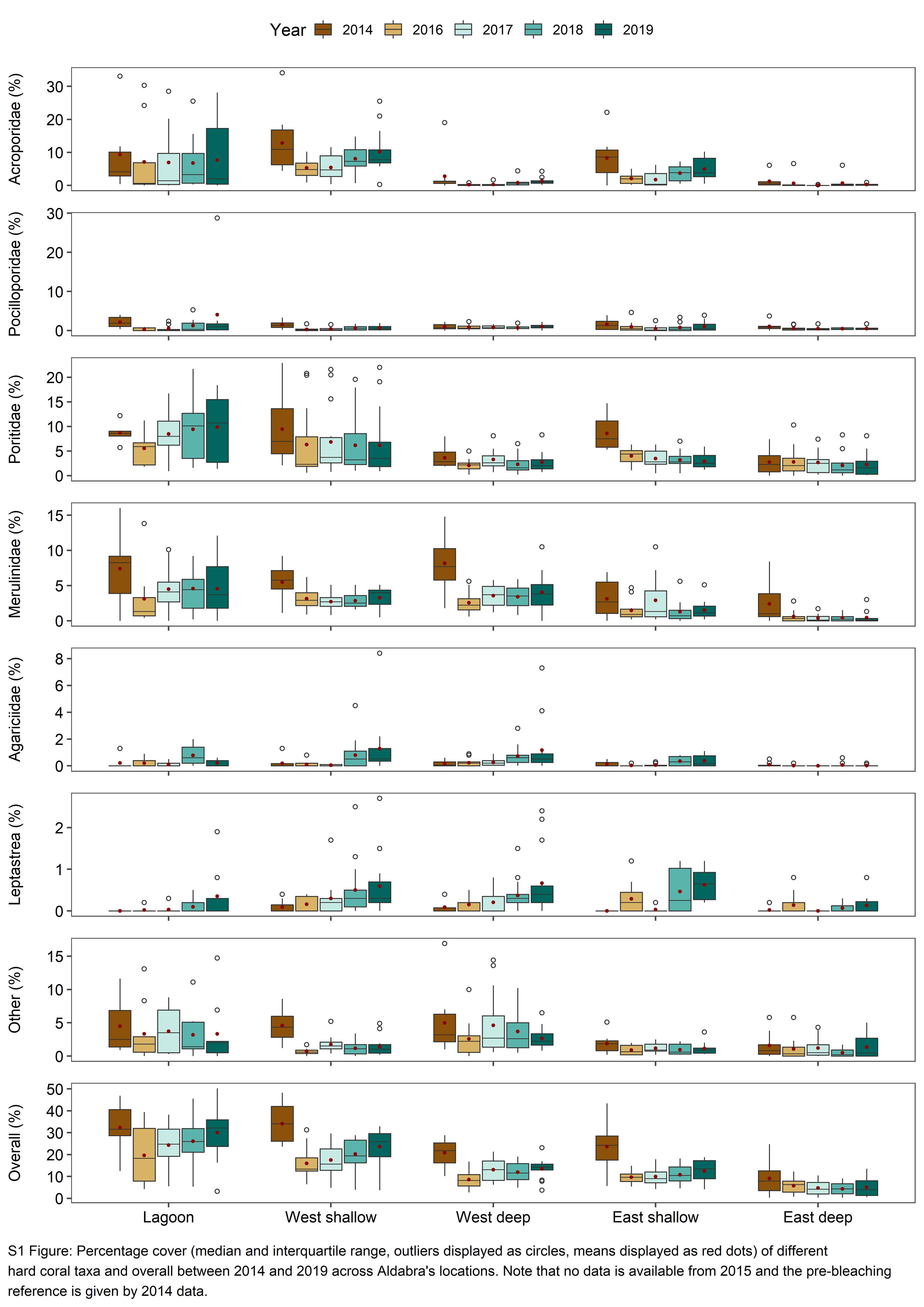

Supplement: S1 Fig — Plots show median and interquartile range with outliers displayed as circles and means displayed as red dots. Note that no data is available from 2015 and the pre–bleaching reference is the 2014 data. (TIF) [file pone.0260516.s001.tif]
