# Supplementary material for: First insights into coral recruit and juvenile abundances at remote Aldabra Atoll, Seychelles
Source: PLoS One. 2021 Dec 7;16(12):e0260516. doi: 10.1371/journal.pone.0260516 (PMC8651144; doi:10.1371/journal.pone.0260516)
Supplement: S2 Fig — The number (percentage) of sites (blue marked values in S5 Table) with coral juvenile abundances that fall within the same range as Aldabra’s reefs in 2019 (16.1–29.9 coral juveniles m-2) or below/above. (DOCX) [file pone.0260516.s002.docx]

**S2 Figure**. **Comparison of Aldabra’s coral juvenile abundance in 2019 with pre-bleaching values at Aldabra and reefs worldwide.** The number (percentage) of sites (blue marked values in S5 Table) with coral juvenile abundances that fall within the same range as Aldabra’s reefs in 2019 (16.1–29.9 coral juveniles m-2) or below/above.
