# Supplementary material for: First insights into coral recruit and juvenile abundances at remote Aldabra Atoll, Seychelles
Source: PLoS One. 2021 Dec 7;16(12):e0260516. doi: 10.1371/journal.pone.0260516 (PMC8651144; doi:10.1371/journal.pone.0260516)
Supplement: S1 Table — Number of transects and quadrats completed at each location and site during each survey year. Lower number of replicates obtained in 2015 due to limited resources. (DOCX) [file pone.0260516.s003.docx]

**S1 Table. Coral juvenile survey replicates.** Number of transects and quadrats completed at each location and site during each survey year. Lower number of replicates obtained in 2015 due to limited resources.

| Location (water depth)  Site number | 2015 | |  | 2016 | |  | 2018 | |  | 2019 | |
| --- | --- | --- | --- | --- | --- | --- | --- | --- | --- | --- | --- |
|  | Trans. | Quad. |  | Trans. | Quad. |  | Trans. | Quad. |  | Trans. | Quad. |
| **West (5 m)** | **12** | **60** |  | **12** | **60** |  | **15** | **75** |  | **15** | **75** |
| 1 | 3 | 15 |  | 3 | 15 |  | 3 | 15 |  | 3 | 15 |
| 2 | 3 | 15 |  | 3 | 15 |  | 3 | 15 |  | 3 | 15 |
| 6 | 3 | 15 |  | 3 | 15 |  | 3 | 15 |  | 3 | 15 |
| 7 | 0 | 0 |  | 0 | 0 |  | 3 | 15 |  | 3 | 15 |
| 8 | 3 | 15 |  | 3 | 15 |  | 3 | 15 |  | 3 | 15 |
| **West (15 m)** | **4** | **20** |  | **15** | **75** |  | **15** | **75** |  | **15** | **75** |
| 1 | 1 | 5 |  | 3 | 15 |  | 3 | 15 |  | 3 | 15 |
| 2 | 1 | 5 |  | 3 | 15 |  | 3 | 15 |  | 3 | 15 |
| 6 | 1 | 5 |  | 3 | 15 |  | 3 | 15 |  | 3 | 15 |
| 7 | 0 | 0 |  | 3 | 15 |  | 3 | 15 |  | 3 | 15 |
| 8 | 1 | 5 |  | 3 | 15 |  | 3 | 15 |  | 3 | 15 |
| **East (5 m)** | **12** | **60** |  | **12** | **60** |  | **12** | **60** |  | **12** | **60** |
| 3 | 3 | 15 |  | 3 | 15 |  | 3 | 15 |  | 3 | 15 |
| 4 | 3 | 15 |  | 3 | 15 |  | 3 | 15 |  | 3 | 15 |
| 5 | 3 | 15 |  | 3 | 15 |  | 3 | 15 |  | 3 | 15 |
| 12 | 3 | 15 |  | 3 | 15 |  | 3 | 15 |  | 3 | 15 |
| **East (15 m)** | **4** | **15** |  | **12** | **60** |  | **12** | **60** |  | **12** | **60** |
| 3 | 1 | 5 |  | 3 | 15 |  | 3 | 15 |  | 3 | 15 |
| 4 | 0 | 0 |  | 3 | 15 |  | 3 | 15 |  | 3 | 15 |
| 5 | 2 | 5 |  | 3 | 15 |  | 3 | 15 |  | 3 | 15 |
| 12 | 1 | 5 |  | 3 | 15 |  | 3 | 15 |  | 3 | 15 |
| **Lagoon (2 m)** | **0** | **0** |  | **9** | **45** |  | **9** | **45** |  | **9** | **45** |
| 9 | 0 | 0 |  | 3 | 15 |  | 3 | 15 |  | 3 | 15 |
| 10 | 0 | 0 |  | 3 | 15 |  | 3 | 15 |  | 3 | 15 |
| 11 | 0 | 0 |  | 3 | 15 |  | 3 | 15 |  | 3 | 15 |
| Trans. = Transect, Quad. = Quadrat | | | | | | | | | | | |
