# Supplementary material for: First insights into coral recruit and juvenile abundances at remote Aldabra Atoll, Seychelles
Source: PLoS One. 2021 Dec 7;16(12):e0260516. doi: 10.1371/journal.pone.0260516 (PMC8651144; doi:10.1371/journal.pone.0260516)
Supplement: S2 Table — Number of coral settlement tiles retrieved per site and survey period. Replicates at the seaward site vary as some tiles were lost due to rough weather. (DOCX) [file pone.0260516.s004.docx]

**S2 Table**. **Coral recruit survey replicates.** Number of coral recruitment tiles retrieved per site and survey period. Replicates at the seaward site vary as some tiles were lost due to rough weather.

| Survey period | Lagoonal site (Site 9) | Seaward site (Site 1) |
| --- | --- | --- |
| Aug–Oct 18 | 12 | 11 |
| Oct–Dec 18 | 12 | 12 |
| Dec 18–Feb 19 | 12 | 14 |
| Feb–Apr 19 | 12 | 14 |
| Apr–Jun 19 | 12 | 10 |
| Jun–Aug 19 | 12 | 9 |
