# Supplementary material for: First insights into coral recruit and juvenile abundances at remote Aldabra Atoll, Seychelles
Source: PLoS One. 2021 Dec 7;16(12):e0260516. doi: 10.1371/journal.pone.0260516 (PMC8651144; doi:10.1371/journal.pone.0260516)
Supplement: S3 Table — Effect of year (2015, 2016, 2018, 2019), location (lagoon, western seaward, eastern seaward) and depth (2 m, 5 m, 15 m) on coral juvenile abundances at Aldabra. Chi-square-value (χ2), degrees of freedom (dF) and p-value obtained from GLMM model comparisons with ANOVA (type II). Significance level: *** p < 0.001; ** p < 0.01; ** p < 0.05; ns = not significant: p > 0.05. (DOCX) [file pone.0260516.s005.docx]

**S3 Table. Change in coral juvenile abundances.** Effect of year (2015, 2016, 2018, 2019), location (lagoon, western seaward, eastern seaward) and depth (2 m, 5 m, 15 m) on coral juvenile abundances at Aldabra. Chi-square-value (χ^2^), degrees of freedom (dF) and p-value obtained from GLMM model comparisons with ANOVA (type II). Significance level: *** *p* < 0.001; ** *p* < 0.01; ** *p* < 0.05; ns = not significant: *p* > 0.05.

| Group | Overall | | | x | Acroporidae | | | x | Pocilloporidae | | | x | Merulinidae | | | x | Agariciidae | | | x | Other | | | x | Poritidae^a^ | | | x | *Leptastrea*^a^ | | |
| --- | --- | --- | --- | --- | --- | --- | --- | --- | --- | --- | --- | --- | --- | --- | --- | --- | --- | --- | --- | --- | --- | --- | --- | --- | --- | --- | --- | --- | --- | --- | --- |
| Error distribution | Poisson | | |  | Neg. binomial | | |  | Poisson | | |  | Poisson | | |  | Poisson | | |  | Poisson | | |  | Poisson | | |  | Poisson | | |
| Fixed factor | χ^2^ | dF | *p* |  | χ^2^ | dF | *p* |  | χ^2^ | dF | *p* |  | χ^2^ | dF | *p* |  | χ^2^ | dF | *p* |  | χ^2^ | dF | *p* |  | χ^2^ | dF | *p* |  | χ^2^ | dF | *p* |
| Year | 247.2 | 3 | *** |  | 95.0 | 3 | *** |  | 43.5 | 3 | *** |  | 33.0 | 3 | *** |  | 40.4 | 3 | *** |  | 134.3 | 3 | *** |  | 94.8 | 3 | *** |  | 73.1 | 3 | * |
| Location | 6.9 | 2 | * |  | 2.9 | 2 | ns |  | 4.0 | 2 | ns |  | 34.1 | 2 | *** |  | 17.8 | 2 | *** |  | 12.9 | 2 | ** |  | not tested | | |  | not tested | | |
| Depth | 4.9 | 1 | * |  | 21.3 | 1 | *** |  | 0.6 | 1 | ns |  | 6.7 | 1 | ** |  | 6.4 | 1 | * |  | 1.2 | 1 | ns |  | –"– | | |  | –"– | | |
| Year:Location | 73.0 | 5 | *** |  | 25.7 | 5 | *** |  | 9.7 | 5 | ns |  | 14.7 | 5 | * |  | 7.4 | 5 | ns |  | 56.3 | 5 | *** |  | –"– | | |  | –"– | | |
| Year:Depth | 1.8 | 3 | ns |  | 7.0 | 3 | ns |  | 2.6 | 3 | ns |  | 7.9 | 3 | * |  | 10.0 | 3 | * |  | 13.6 | 3 | ** |  | –"– | | |  | –"– | | |
| Location:Depth | 1.5 | 1 | ns |  | 1.0 | 1 | ns |  | 0.3 | 1 | ns |  | 0.3 | 1 | ns |  | 0.0 | 1 | ns |  | 3.0 | 1 | ns |  | –"– | | |  | –"– | | |
| Year:Location:Depth | 15.3 | 3 | ** |  | 4.9 | 3 | ns |  | 11.4 | 3 | *** |  | 9.4 | 3 | * |  | 13.3 | 3 | ** |  | 5.9 | 3 | ns |  | –"– | | |  | –"– | | |
| ^a^ models including interacting fixed factors had poor model fit and only differences across years were test (see methods) | | | | | | | | | | | | | | | | | | | | | | | | | | | | | | | |
