# Supplementary material for: First insights into coral recruit and juvenile abundances at remote Aldabra Atoll, Seychelles
Source: PLoS One. 2021 Dec 7;16(12):e0260516. doi: 10.1371/journal.pone.0260516 (PMC8651144; doi:10.1371/journal.pone.0260516)
Supplement: S4 Table — Effect of location (seaward western site: Site 1, lagoonal site: Site 9) and time period (Aug–Oct 2018, Oct–Dec 2018, Dec 2018–Feb 2019, Feb–Apr 2019, Apr–Jun 2019, Jun–Aug 2019) on coral recruit abundances on settlement tiles. Chi-square-value (χ2), degrees of freedom (dF) and p-value obtained from GLMM model comparisons with ANOVA (type II). Results of the pairwise tests were derived from post-hoc analysis based on least square means with Bonferroni adjustment. Significance level: *** p < 0.001; ** p < 0.01. (DOCX) [file pone.0260516.s006.docx]

**S4 Table**. **Difference in coral recruit abundances.** Effect of location (seaward western site: Site 1, lagoonal site: Site 9) and time period (Aug–Oct 2018, Oct–Dec 2018, Dec 2018–Feb 2019, Feb–Apr 2019, Apr–Jun 2019, Jun–Aug 2019) on coral recruit abundances on settlement tiles. Chi-square-value (χ^2^), degrees of freedom (dF) and *p*-value obtained from GLMM model comparisons with ANOVA (type II). Results of the pairwise tests were derived from post-hoc analysis based on least square means with Bonferroni adjustment. Significance level: *** *p* < 0.001; ** *p* < 0.01; n.s. = not significant.

|  | GLMM model comparison ANOVA (type II) | | | Pairwise test | |
| --- | --- | --- | --- | --- | --- |
| Fixed factor | χ^2^ | dF | *p* | t-ratio | *p* |
| Location | 181.4 | 1 | *** |  |  |
| Period | 89.0 | 5 | *** |  |  |
| Location:Period | 16.7 | 5 | ** |  |  |
| *Aug – Oct, Site 1 vs 9* |  |  |  | -6.7 | *** |
| *Oct – Dec, Site 1 vs 9* |  |  |  | -8.2 | *** |
| *Dec – Feb, Site 1 vs 9* |  |  |  | -6.6 | *** |
| *Feb – Apr, Site 1 vs 9* |  |  |  | -6.8 | *** |
| *Apr – Jun, Site 1 vs 9* |  |  |  | -4.9 | *** |
| *Jun – Aug, Site 1 vs 9* |  |  |  | -0.9 | n.s. |
