# Supplementary material for: First insights into coral recruit and juvenile abundances at remote Aldabra Atoll, Seychelles
Source: PLoS One. 2021 Dec 7;16(12):e0260516. doi: 10.1371/journal.pone.0260516 (PMC8651144; doi:10.1371/journal.pone.0260516)
Supplement: S5 Table — A: Studies reporting coral juvenile abundances before and/or after a bleaching event. B: Studies reporting coral juvenile abundance without referring to a bleaching event. Values in blue are those included in S2 Fig (comparison of Aldabra’s coral juvenile abundance in 2019 to pre-bleaching values at Aldabra and reefs worldwide). ‘Nr. years studied’ refers to number of years data was collected within each study. (DOCX) [file pone.0260516.s007.docx]

**S5 Table.** **Coral juvenile abundances (per m^2^) reported from 106 reefs at 11 locations worldwide.** A: Studies reporting coral juvenile abundances before and/or after a bleaching event. B: Studies reporting coral juvenile abundance without referring to a bleaching event. Values in blue are those included in S2 Figure (comparison of Aldabra’s coral juvenile abundance in 2019 to pre-bleaching values at Aldabra and reefs worldwide). ‘Nr. years studied’ refers to number of years data was collected within each study.

| **A** | | Pre-bleaching | | |  | First record post-bleaching | | |  | Last record post-bleaching | | |  | Nr. years studied |  | |
| --- | --- | --- | --- | --- | --- | --- | --- | --- | --- | --- | --- | --- | --- | --- | --- | --- |
| Location (bleaching event) | | Year | Mean | SE | x | Year | Mean | SE | % change^a^ | Year | Mean | SE | % change^b^ |  | Level of protection | Source and notes |
|  | |  |  |  |  |  |  |  |  |  |  |  |  |  |  |  |
| **Aldabra Atoll, Seychelles (2015/16)** | | 2015 | 14.1 | 1.2 |  | 2016 | 7.4 | 0.5 | -48 | 2019 | 22.4 | 1.2 | 67 | 4 | Special Reserve (1981) | This study |
| Lagoon | | ⸺"⸺ | n.a. | n.a. |  | ⸺"⸺ | 7.7 | 1.6 | n.a. | ⸺"⸺ | 29.9 | 5.3 | 74 | ⸺"⸺ | ⸺"⸺ |  |
| West (5 m) | | ⸺"⸺ | 20.6 | 1.9 |  | ⸺"⸺ | 8.3 | 1.0 | -60 | ⸺"⸺ | 21.4 | 1.9 | 61 | ⸺"⸺ | ⸺"⸺ |  |
| East (5 m) | | ⸺"⸺ | 8.3 | 0.8 |  | ⸺"⸺ | 7.9 | 1.0 | -5 | ⸺"⸺ | 24.1 | 2.6 | 67 | ⸺"⸺ | ⸺"⸺ |  |
| West (15 m) | | ⸺"⸺ | 14.2 | 5.1 |  | ⸺"⸺ | 7.7 | 0.8 | -46 | ⸺"⸺ | 22.3 | 1.8 | 65 | ⸺"⸺ | ⸺"⸺ |  |
| East (15 m) | | ⸺"⸺ | 11.5 | 2.7 |  | ⸺"⸺ | 5.5 | 1.0 | -52 | ⸺"⸺ | 16.6 | 1.9 | 67 | ⸺"⸺ | ⸺"⸺ |  |
|  | |  |  |  |  |  |  |  |  |  |  |  |  |  |  |  |
| **Aldabra Atoll, Seychelles (1998/99)** | |  |  |  |  | 2001 | 6.0 | 0.8 | n.a. | 2005 | 5.9 | 0.9 | -2 | 4 | Special Reserve (1981) | Stobart et al. (2002); Buckley et al. (2005)  Values obtained by averaging site level data given in references |
| Lagoon | |  |  |  |  | ⸺"⸺ | 7.4 | 1.9 | ⸺"⸺. | n.a. |  |  | n.a. | ⸺"⸺ | ⸺"⸺ |  |
| West (6 m) | |  |  |  |  | ⸺"⸺ | 4.5 | 0.8 | ⸺"⸺ | ⸺"⸺ |  |  | ⸺"⸺ | ⸺"⸺ | ⸺"⸺ |  |
| East (6 m) | |  |  |  |  | ⸺"⸺ | 8.2 | 0.4 | ⸺"⸺ | ⸺"⸺ |  |  | ⸺"⸺ | ⸺"⸺ | ⸺"⸺ |  |
| West (10 m) | |  |  |  |  | ⸺"⸺ | 6.7 | 1.8 | ⸺"⸺ | 2005 | 4.4 | 0.7 | -54 | ⸺"⸺ | ⸺"⸺ |  |
| East (10 m) | |  |  |  |  | ⸺"⸺ | 7.6 | 1.0 | ⸺"⸺ | ⸺"⸺ | 7.4 | 1.0 | -4 | ⸺"⸺ | ⸺"⸺ |  |
| West (20 m) | |  |  |  |  | ⸺"⸺ | 4.1 | 1.0 | ⸺"⸺ | n.a. |  |  | ⸺"⸺ | ⸺"⸺ | ⸺"⸺ |  |
| East (20 m) | |  |  |  |  | ⸺"⸺ | 3.7 | 1.6 | ⸺"⸺ | ⸺"⸺ |  |  | ⸺"⸺ | ⸺"⸺ | ⸺"⸺ |  |
|  | |  |  |  |  |  |  |  |  |  |  |  |  |  |  |  |
| **Inner Islands, Seychelles (2016)** | | 2011 | 11.9 | 2.0 |  | 2017 | 3.1 | 0.7 | -74 |  |  |  |  | 3 |  | Dajka et al. (2019)  Reef level values obtained with approval from J. Dajka and K. Chong-Seng (pers. comm, July 2020)  Protection: Jennings et al. (2020) |
| Mahe East Carbonate | | ⸺"⸺ | 4.3 | 1.2 |  | ⸺"⸺ | 1.2 | 0.6 | -72 |  |  |  |  | ⸺"⸺ |  |  |
| Mahe East Granite | | ⸺"⸺ | 5.2 | 1.1 |  | ⸺"⸺ | 0.0 | 0.0 | -100 |  |  |  |  | ⸺"⸺ |  |  |
| Mahe East Patch | | ⸺"⸺ | 15.0 | 1.7 |  | ⸺"⸺ | 6.2 | 2.0 | -59 |  |  |  |  | ⸺"⸺ |  |  |
| Mahe North West Carbonate | | ⸺"⸺ | 12.3 | 3.5 |  | ⸺"⸺ | 0.9 | 0.4 | -92 |  |  |  |  | ⸺"⸺ | No-take MNP (1979) |  |
| Mahe North West Granite | | ⸺"⸺ | 37.9 | 4.1 |  | ⸺"⸺ | 8.3 | 2.3 | -78 |  |  |  |  | ⸺"⸺ |  |  |
| Mahe North West Patch | | ⸺"⸺ | 10.6 | 1.7 |  | ⸺"⸺ | 3.1 | 0.9 | -71 |  |  |  |  | ⸺"⸺ |  |  |
| Mahe St. Anne Carbonate | | ⸺"⸺ | 15.1 | 2.8 |  | ⸺"⸺ | 1.0 | 0.7 | -93 |  |  |  |  | ⸺"⸺ | No-take MNP (1973) |  |
| Mahe St. Anne Granite | | ⸺"⸺ | 8.2 | 1.4 |  | ⸺"⸺ | 6.2 | 1.8 | -25 |  |  |  |  | ⸺"⸺ | ⸺"⸺ |  |
| Mahe St. Anne Patch | | ⸺"⸺ | 15.0 | 2.1 |  | ⸺"⸺ | 0.2 | 0.2 | -98 |  |  |  |  | ⸺"⸺ | ⸺"⸺ |  |
| Mahe West Carbonate | | ⸺"⸺ | 9.2 | 1.4 |  | ⸺"⸺ | 1.2 | 0.6 | -87 |  |  |  |  | ⸺"⸺ |  |  |
| Mahe West Granite | | ⸺"⸺ | 15.9 | 2.6 |  | ⸺"⸺ | 9.1 | 2.2 | -43 |  |  |  |  | ⸺"⸺ |  |  |
|  | |  |  |  |  |  |  |  |  |  |  |  |  |  | *(continued on next page)* | |
|  |  |  |  |  |  |  |  |  |  |  |  |  |  |  |  |  |
| **A** | | Pre-bleaching | | |  | First record post-bleaching | | |  | Last record post-bleaching | | |  | Nr. years studied |  | |
| Location (bleaching event) | | Year | Mean | SE | x | Year | Mean | SE | % change^a^ | Year | Mean | SE | % change^b^ |  | Level of protection | Source |
|  | |  |  |  |  |  |  |  |  |  |  |  |  |  |  |  |
| Mahe West Patch | | ⸺"⸺ | 13.0 | 2.8 |  | ⸺"⸺ | 0.5 | 0.3 | -96 |  |  |  |  | 3 |  |  |
| Praslin North East Carbonate | | ⸺"⸺ | 1.9 | 0.7 |  | ⸺"⸺ | 0.3 | 0.2 | -84 |  |  |  |  | ⸺"⸺ |  |  |
| Praslin North East Granite | | ⸺"⸺ | 16.9 | 2.7 |  | ⸺"⸺ | 4.0 | 0.8 | -76 |  |  |  |  | ⸺"⸺ | No-take MNP (1979) |  |
| Praslin North East Patch | | ⸺"⸺ | 4.1 | 0.9 |  | ⸺"⸺ | 2.6 | 0.6 | -38 |  |  |  |  | ⸺"⸺ | ⸺"⸺ |  |
| Praslin South West Carbonate | | ⸺"⸺ | 0.7 | 0.4 |  | ⸺"⸺ | 1.6 | 0.8 | 135 |  |  |  |  | ⸺"⸺ |  |  |
| Praslin South West Granite | | ⸺"⸺ | 9.5 | 1.3 |  | ⸺"⸺ | 6.0 | 1.9 | -37 |  |  |  |  | ⸺"⸺ |  |  |
| Praslin South West Patch | | ⸺"⸺ | 18.7 | 3.3 |  | ⸺"⸺ | 3.8 | 1.1 | -80 |  |  |  |  | ⸺"⸺ |  |  |
|  | |  |  |  |  |  |  |  |  |  |  |  |  |  |  |  |
| **Lizard Island, Australia (2016)** | | 2015 | 6.0 | 1.2 |  | 2016 | 5.4 | 0.7 | -10 |  |  |  |  | 2 | National Park (1939) | Álvarez-Noriega et al. (2018)  Values estimated from Fig. A1 in supplement  Protection: Queensland Government  https://parks.des.qld.gov.au/parks/lizard-island/about#management |
| North Reef 1 | | ⸺"⸺ | 10.6 | 0.5 |  | ⸺"⸺ | 10.5 | 0.3 | -1 |  |  |  |  | ⸺"⸺ | ⸺"⸺ |  |
| North Reef 2 | | ⸺"⸺ | 3.2 | 1.0 |  | ⸺"⸺ | 5.4 | 0.6 | 69 |  |  |  |  | ⸺"⸺ | ⸺"⸺ |  |
| North Reef 3 | | ⸺"⸺ | 15.0 | 1.0 |  | ⸺"⸺ | 8.9 | 0.6 | -41 |  |  |  |  | ⸺"⸺ | ⸺"⸺ |  |
| Gnarly Tree | | ⸺"⸺ | 9.8 | 1.0 |  | ⸺"⸺ | 8.9 | 1.0 | -9 |  |  |  |  | ⸺"⸺ | ⸺"⸺ |  |
| North of Paradise | | ⸺"⸺ | 8.4 | 2.1 |  | ⸺"⸺ | 7.6 | 1.3 | -10 |  |  |  |  | ⸺"⸺ | ⸺"⸺ |  |
| No Man’s Land | | ⸺"⸺ | 9.0 | 2.2 |  | ⸺"⸺ | 6.8 | 1.1 | -24 |  |  |  |  | ⸺"⸺ | ⸺"⸺ |  |
| Easter Point | | ⸺"⸺ | 10.2 | 1.3 |  | ⸺"⸺ | 7.6 | 1.2 | -25 |  |  |  |  | ⸺"⸺ | ⸺"⸺ |  |
| Lizard Head | | ⸺"⸺ | 8.6 | 2.0 |  | ⸺"⸺ | 8.1 | 0.6 | -6 |  |  |  |  | ⸺"⸺ | ⸺"⸺ |  |
| Southeast 1 | | ⸺"⸺ | 3.0 | 1.3 |  | ⸺"⸺ | 5.4 | 0.5 | 80 |  |  |  |  | ⸺"⸺ | ⸺"⸺ |  |
| South Island | | ⸺"⸺ | 8.4 | 1.3 |  | ⸺"⸺ | 5.7 | 0.6 | -32 |  |  |  |  | ⸺"⸺ | ⸺"⸺ |  |
| Lagoon 1 | | ⸺"⸺ | 3.0 | 0.5 |  | ⸺"⸺ | 4.1 | 0.5 | 37 |  |  |  |  | ⸺"⸺ | ⸺"⸺ |  |
| Trimodal 1 | | ⸺"⸺ | 4.4 | 1.6 |  | ⸺"⸺ | 2.4 | 0.5 | -45 |  |  |  |  | ⸺"⸺ | ⸺"⸺ |  |
| Lagoon 2 | | ⸺"⸺ | 1.6 | 0.6 |  | ⸺"⸺ | 3.2 | 0.7 | 100 |  |  |  |  | ⸺"⸺ | ⸺"⸺ |  |
| Horseshoe | | ⸺"⸺ | 5.4 | 1.1 |  | ⸺"⸺ | 1.9 | 0.5 | -65 |  |  |  |  | ⸺"⸺ | ⸺"⸺ |  |
| Vickis | | ⸺"⸺ | 2.2 | 0.5 |  | ⸺"⸺ | 3.8 | 1.0 | 73 |  |  |  |  | ⸺"⸺ | ⸺"⸺ |  |
| Corner Beach | | ⸺"⸺ | 3.4 | 1.0 |  | ⸺"⸺ | 2.7 | 0.7 | -21 |  |  |  |  | ⸺"⸺ | ⸺"⸺ |  |
| Osprey | | ⸺"⸺ | 1.0 | 0.5 |  | ⸺"⸺ | 2.7 | 0.8 | 170 |  |  |  |  | ⸺"⸺ | ⸺"⸺ |  |
| Resort | | ⸺"⸺ | 5.0 | 1.5 |  | ⸺"⸺ | 5.4 | 0.6 | 8 |  |  |  |  | ⸺"⸺ | ⸺"⸺ |  |
| Cooks Path | | ⸺"⸺ | 4.4 | 2.1 |  | ⸺"⸺ | 3.0 | 0.7 | -32 |  |  |  |  | ⸺"⸺ | ⸺"⸺ |  |
| Turtle | | ⸺"⸺ | 2.4 | 0.5 |  | ⸺"⸺ | 3.2 | 0.6 | 33 |  |  |  |  | ⸺"⸺ | ⸺"⸺ |  |
|  | |  |  |  |  |  |  |  |  |  |  |  |  |  | *(continued on next page)* | |
|  | |  |  |  |  |  |  |  |  |  |  |  |  |  |  | |
|  | |  |  |  |  |  |  |  |  |  |  |  |  |  |  |  |
|  |  |  |  |  |  |  |  |  |  |  |  |  |  |  |  |  |
| **A** | | Pre-bleaching | | |  | First record post-bleaching | | |  | Last record post-bleaching | | |  | Nr. years studied |  | |
| Location (bleaching event) | | Year | Mean | SE | x | Year | Mean | SE | % change^a^ | Year | Mean | SE | % change^b^ |  | Level of protection | Source |
|  | |  |  |  |  |  |  |  |  |  |  |  |  |  |  |  |
| **Huvadhoo Atoll, Maldives (2016)** | |  |  |  |  | 2017 | 4 | 6.4* | n.a. |  |  |  |  | 1 | None | Perry and Morgan (2017) |
| Mahutigala (< 5m depth) | |  |  |  |  | ⸺"⸺ | 5.9 | 12.0* | ⸺"⸺ |  |  |  |  | ⸺"⸺ |  |  |
| Kandahalagala | |  |  |  |  | ⸺"⸺ | 3 | 3.5* | ⸺"⸺ |  |  |  |  | ⸺"⸺ |  |  |
| Kodehutigalaa | |  |  |  |  | ⸺"⸺ | 3.5 | 3.7* | ⸺"⸺ |  |  |  |  | ⸺"⸺ |  |  |
| Kadumaigala | |  |  |  |  | ⸺"⸺ | 2.7 | 4.6* | ⸺"⸺ |  |  |  |  | ⸺"⸺ |  |  |
| Kafigahla | |  |  |  |  | ⸺"⸺ | 5 | 8.0* | ⸺"⸺ |  |  |  |  | ⸺"⸺ |  |  |
|  | |  |  |  |  |  |  |  |  |  |  |  |  |  |  |  |
| **Palau (1998/99)** | |  |  |  |  | 2001 | 4.9 | 1.1 | n.a. | 2004 | 3.8 | 0.8 | -29 | 3 | No-take MPAs (individual sites,  1976–2005) | Golbuu et al. (2007)  Protection: Friedlander et al. (2017), Golbuu et al. (2005) |
| West Exposed (3 m) | |  |  |  |  | ⸺"⸺ | 7.5 | 1.0 | ⸺"⸺ | ⸺"⸺ | 4 | 0.5 | -88 | ⸺"⸺ |  |  |
| East Exposed (3 m) | |  |  |  |  | ⸺"⸺ | 4.5 | 1.0 | ⸺"⸺ | ⸺"⸺ | 6 | 0.9 | 25 | ⸺"⸺ |  |  |
| Patch (3 m) | |  |  |  |  | ⸺"⸺ | 5.5 | 1.5 | ⸺"⸺ | ⸺"⸺ | 2.5 | 0.5 | -120 | ⸺"⸺ |  |  |
| Bay (3 m) | |  |  |  |  | ⸺"⸺ | 4 | 1.0 | ⸺"⸺ | ⸺"⸺ | 1.5 | 0.5 | -167 | ⸺"⸺ |  |  |
| West Exposed (10 m) | |  |  |  |  | ⸺"⸺ | 7.5 | 1.0 | ⸺"⸺ | ⸺"⸺ | 4 | 0.5 | -88 | ⸺"⸺ |  |  |
| East Exposed (10 m) | |  |  |  |  | ⸺"⸺ | 4 | 0.9 | ⸺"⸺ | ⸺"⸺ | 4 | 1 | 0 | ⸺"⸺ |  |  |
| Patch (10 m) | |  |  |  |  | ⸺"⸺ | 3 | 1.0 | ⸺"⸺ | ⸺"⸺ | 4 | 1.1 | 25 | ⸺"⸺ |  |  |
| Bay (10 m) | |  |  |  |  | ⸺"⸺ | 3 | 1.0 | ⸺"⸺ | ⸺"⸺ | 4 | 1 | 25 | ⸺"⸺ |  |  |
|  | |  |  |  |  |  |  |  |  |  |  |  |  |  |  | *(continued on next page)* |
| ^a^ Percentage difference in coral juvenile abundance between pre-bleaching value and first record post-bleaching  ^b^ Percentage difference in coral juvenile abundance between first and last record post-bleaching  * SD not SE  n.a. = not applicable  MPA = Marine Protected Area | | | | | | | | | | | |  |  |  |  | |

| **S5 Table** (continued) | | | | | | | | |
| --- | --- | --- | --- | --- | --- | --- | --- | --- |
| **B** |  |  |  |  |  | Nr. years studied | |  |
| Location | Year | Mean | SE |  | Level of protection |  |  | Source and notes |
|  |  |  |  |  |  |  | |  |
| **Central atolls Maldives** | 2007 | 28.2 | 1.1 |  | None | 2 | | Cardini et al. (2012) |
| Lagoon (5-6 m ) | ⸺"⸺ | 23.1 | 1.2 |  |  | ⸺"⸺ | |  |
| Lagoon (10-12 m ) | ⸺"⸺ | 23.0 | 1.0 |  |  | ⸺"⸺ | |  |
| Lagoon (16-18 m ) | ⸺"⸺ | 20.8 | 1.0 |  |  | ⸺"⸺ | |  |
| Ocean facing reef (5-6 m ) | ⸺"⸺ | 38.2 | 1.1 |  |  | ⸺"⸺ | |  |
| Ocean facing reef (10-12 m ) | ⸺"⸺ | 34.3 | 1.5 |  |  | ⸺"⸺ | |  |
| Ocean facing reef (16-18 m ) | ⸺"⸺ | 29.5 | 0.9 |  |  | ⸺"⸺ | |  |
|  |  |  |  |  |  |  | |  |
| **Moorea, French Polynesia** | 2003 | 7.7 | 1.3 |  | None (at that time) | 1 | | Penin et al. (2007) |
| Vaipahu (6 m) | ⸺"⸺ | 6.8 | 1.0 |  |  | ⸺"⸺ | |  |
| Vaipahu (12 m) | ⸺"⸺ | 7.3 | 0.5 |  |  | ⸺"⸺ | |  |
| Vaipahu (18 m) | ⸺"⸺ | 10.5 | 1.2 |  |  | ⸺"⸺ | |  |
| Tiahura (6 m) | ⸺"⸺ | 7.1 | 0.8 |  |  | ⸺"⸺ | |  |
| Tiahura (12 m) | ⸺"⸺ | 8.1 | 1.2 |  |  | ⸺"⸺ | |  |
| Tiahura (18 m) | ⸺"⸺ | 12.3 | 3.8 |  |  | ⸺"⸺ | |  |
| Haapiti (6 m) | ⸺"⸺ | 3.7 | 1.0 |  |  | ⸺"⸺ | |  |
| Haapiti (12 m) | ⸺"⸺ | 6.1 | 1.2 |  |  | ⸺"⸺ | |  |
| Haapiti (18 m) | ⸺"⸺ | 7.7 | 0.8 |  |  | ⸺"⸺ | |  |
|  |  |  |  |  |  |  | |  |
| **Grande Terre, New Caledonia** | 2008 | 9.4 | 1.1 |  | None (at that time) | 3 | | Adjeroud et al. (2019) |
| SM1 | ⸺"⸺ | 6.5 | 2.5 |  |  | ⸺"⸺ | |  |
| SM2 | ⸺"⸺ | 4.0 | 0.8 |  |  | ⸺"⸺ | |  |
| RA1 | ⸺"⸺ | 9.5 | 2.5 |  |  | ⸺"⸺ | |  |
| RA2 | ⸺"⸺ | 3.0 | 0.7 |  |  | ⸺"⸺ | |  |
| MA1 | ⸺"⸺ | 5.5 | 0.5 |  |  | ⸺"⸺ | |  |
| MA2 | ⸺"⸺ | 3.0 | 0.8 |  |  | ⸺"⸺ | |  |
| MS1 | ⸺"⸺ | 4.8 | 0.9 |  |  | ⸺"⸺ | |  |
| MS2 | ⸺"⸺ | 17.0 | 0.5 |  |  | ⸺"⸺ | |  |
| BA1 | ⸺"⸺ | 20.0 | 0.8 |  |  | ⸺"⸺ | |  |
| BA2 | ⸺"⸺ | 20.2 | 0.8 |  |  | ⸺"⸺ | |  |
|  |  |  |  |  |  |  | |  |
| **Tongatapu, Tonga** | 2006 | 5.5 | 1.0 |  | None (at that time) | 1 | | Adjeroud et al (2013) |
| S1 (2 -3 m) | ⸺"⸺ | 8.0 | 1.8 |  |  | ⸺"⸺ | |  |
| S2 | ⸺"⸺ | 4.8 | 0.5 |  |  | ⸺"⸺ | |  |
| S3 | ⸺"⸺ | 8.5 | 1.5 |  |  | ⸺"⸺ | |  |
| S4 | ⸺"⸺ | 3.8 | 1.5 |  |  | ⸺"⸺ | |  |
| S5 | ⸺"⸺ | 6.5 | 1.0 |  |  | ⸺"⸺ | |  |
| S6 | ⸺"⸺ | 1.6 | 0.1 |  |  | ⸺"⸺ | |  |
| S7 | ⸺"⸺ | 10.3 | 2.0 |  |  | ⸺"⸺ | |  |
| S8 | ⸺"⸺ | 5.0 | 0.5 |  |  | ⸺"⸺ | |  |
| S9 | ⸺"⸺ | 0.7 | 0.1 |  |  | ⸺"⸺ | |  |
| S10 | ⸺"⸺ | 5.8 | 0.5 |  |  | ⸺"⸺ | |  |
|  |  |  |  |  |  | | *(continued on next page)* | |
|  |  |  |  |  |  |  | |  |
| **S5 Table** (continued) | | | | | | | | |
| **B** |  |  |  |  |  | Nr. years studied | |  |
| Location | Year | Mean | SE |  | Level of protection |  |  | Source and notes |
|  |  |  |  |  |  |  | |  |
| **Vamizi Island, Mozambique** | 2013 | 17.8 | 11.6* |  |  | 1 | | Sola et al. (2015) |
| S1 (5 - 9 m) | ⸺"⸺ | 17.3 | 12.2* |  | No-take MR (2006) | ⸺"⸺ | | Protection: da Silva et al (2015) |
| S2 (5 - 9 m) | ⸺"⸺ | 25.0 | 18.2* |  | ⸺"⸺ | ⸺"⸺ | |  |
| S3 (5 - 9 m) | ⸺"⸺ | 11.0 | 4.4* |  |  | ⸺"⸺ | |  |
|  |  |  |  |  |  |  | |  |
| **United Arab Emirates** | 2012 | 3.8 | 0.4 |  | None | 1 | | Pratchett et al. (2017) |
| Delma | ⸺"⸺ | 2.5 | 0.4 |  |  | ⸺"⸺ | |  |
| Saadiyat | ⸺"⸺ | 5 | 0.5 |  |  | ⸺"⸺ | |  |
| Ra Ghanada | ⸺"⸺ | 5.5 | 1 |  |  | ⸺"⸺ | |  |
| Dibba Rock | ⸺"⸺ | 2.3 | 0.3 |  |  | ⸺"⸺ | |  |
| Al Aqua | ⸺"⸺ | 3.9 | 0.6 |  |  | ⸺"⸺ | |  |
| * SD not SE  MR = marine reserve |  |  |  |  |  |  | |  |
|  |  |  |  |  |  |  | |  |

# Literature cited in S5 Table

Adjeroud M, Briand MJ, Kayal M, Dumas P (2013) Coral assemblages in Tonga: Spatial patterns, replenishment capacities, and implications for conservation strategies. Environ Monit Assess 185:5763–5773

Adjeroud M, Poisson E, Peignon C, Penin L, Kayal M (2019) Spatial Patterns and Short-term Changes of Coral Assemblages Along a Cross-shelf Gradient in the Southwestern Lagoon of New Caledonia. Diversity 11:21

Álvarez-Noriega M, Baird AH, Bridge TCL, Dornelas M, Fontoura L, Pizarro O, Precoda K, Torres-Pulliza D, Woods RM, Zawada K, Madin JS (2018) Contrasting patterns of changes in abundance following a bleaching event between juvenile and adult scleractinian corals. Coral Reefs 37:527–532

Buckley R, Downing N, Stobart B, Teleki K (2005) Aldabra Marine Programme Phase VI. Aldabra: Report on the 2005 Aldabra Marine Programme Research. Cambridge Coastal Research Unit. Department of Geography, University of Cambridge

Cardini U, Chiantore M, Lasagna R, Morri C, Bianchi CN (2012) Size-structure patterns of juvenile hard corals in the Maldives. J Mar Biol Assoc U K 92:1335–1339

Dajka JC, Wilson SK, Robinson JPW, Chong-Seng K, Harris A, Graham N (2019) Uncovering drivers of juvenile coral abundance following mass bleaching. Coral Reefs 38: 637–649

Friedlander AM, Golbuu Y, Ballesteros E, Caselle JE, Gouezo M, Olsudong D, Sala E (2017) Size, age, and habitat determine effectiveness of Palau’s Marine Protected Areas. PLoS One 12:1–18

Golbuu Y, Bauman A, Kuartei J, Victor S (2005) The State of Coral Reef Ecosystems of Palau. In: The State of Coral Reef Ecosystems of the United States and Pacific Freely Associated States: 2005. NOAA Technical Memorandum NOS NCCOS 11. Waddell JE (ed) NOAA/NCCOS, Silver Spring, p 488–505

Golbuu Y, Victor S, Penland L, Idip D, Emaurois C, Okaji K, Yukihira H, Iwase A, van Woesik R (2007) Palau’s coral reefs show differential habitat recovery following the 1998-bleaching event. Coral Reefs 26:319–332

Jennings S, Marshall SS, Cuet P, Naim O (2000) Chapter 13. The Seychelles. In: Coral Reefs of the Indian Ocean. Their Ecology and Conservation. McClanahan TR, Sheppard CRC, Obura DO (eds) Oxford University Press, New York, p 383–410

Penin L, Adjeroud M, Pratchett MS, Hughes T (2007) Spatial distribution of juvenile and adult corals around Moorea (French Polynesia): Implications for population regulation. Bull. Mar. Sci. 80:379–389

Perry CT, Morgan KM (2017) Post-bleaching coral community change on southern Maldivian reefs: is there potential for rapid recovery? Coral Reefs 36:1189–1194

Pratchett M, Baird AH, Bauman AG, Burt J (2017) Abundance and composition of juvenile corals reveals divergent trajectories for coral assemblages across the United Arab Emirates. Mar Pollut Bull 114:1031–1035

Da Silva IM, Hill N, Shimadzu H, Soares AMVM, Dornelas M (2015) Spillover effects of a community-managed marine reserve. PLoS One 10:e0111774

Sola E, Marques da Silva I, Glassom D (2015) Spatio-temporal patterns of coral recruitment at Vamizi Island, Quirimbas Archipelago, Mozambique. African J Mar Sci 37:557–565

Stobart B, Buckley R, Downing N, Callow M, LeClair L, Teleki K (2002) Aldabra Marine Programme Phase III. Aldabra: Extending the Survey Eastward. Cambridge Coastal Research Unit. Department of Geography, University of Cambridge
