# Supplementary material for: First insights into coral recruit and juvenile abundances at remote Aldabra Atoll, Seychelles
Source: PLoS One. 2021 Dec 7;16(12):e0260516. doi: 10.1371/journal.pone.0260516 (PMC8651144; doi:10.1371/journal.pone.0260516)
Supplement: Attachment — Submitted filename: Review_KoesterEtAl_PLoSONE_JCD.docx [file pone.0260516.s012.docx]

REVIEWER REPORT FOR THE AUTHORS

PLoS ONE

Review of Koester, Ford, Ferse, Migani, Bunbury, Sanchez and Wild “First insights into coral recruitment and juvenile abundances at remote Aldabra Atoll, Seychelles”

The study of Koester, Ford, Ferse, Migani, Bunbury, Sanchez and Wild assessed coral recruitment in the Aldabra Atoll of the Outer Seychelles by looking at two vital life cycle stages, coral recruits and coral juveniles.

Koester et al. give interesting and important insights into the impact of the 2016 mass coral bleaching event on Aldabra Atoll and the recruitment dynamics after it. They also assess more limited datasets before the event. The reported coral recovery of the remote atoll is remarkably rapid and indicates the potential of corals to respond to bleaching events with reduced presence of human stressors. This is an important insight to see published. Koester et al.’s interpretations of the findings are on point; I especially enjoyed their interpretations of their results and the corresponding hypotheses as to why they could be seen. I have minor suggestions for improvements.

The manuscript is carefully prepared and well-written. The most important of my improvement suggestions are regarding the structure of introduction and discussion. Some sentences are very long as well, I have marked the ones that definitely need breaking up. Please find specific comments below.

Specific comments

Introduction:

General – Sometimes, ‘recruit’ and ‘recruitment’ get used interchangeably in the text. For instance, in line 200: “As the coral recruitment dataset may have an excess of zeros”. Should this not be the ‘coral recruit dataset’?

To my knowledge, ‘coral recruitment’ is the replenishment of the local adult population by new individuals from within or outside an existing population (Hughes et al. 2010). I always understood this as coral juveniles being included in the recruitment process. Therefore, I would be careful when treating the words interchangeably.

Hughes TP, Graham NAJ, Jackson JBC, Mumby PJ, Steneck RS (2010) Rising to the challenge of sustaining coral reef resilience. Trends in Ecology & Evolution 25:633–642

General – I do not think you need to introduce recruitment dynamic topics like rubble and CCA in the introduction, since neither are picked up in the rest of the manuscript. I would delete the phrases regarding both CCA and rubble.

Methods:

L136 – Is the sentence regarding approval necessary in a manuscript? I have not had the need to include it yet.

L145 – I think you might need a reference for that fragmented colonies statement.

L148-153 – Very long sentence, needs breaking up.

L198 – The comma after “i.e.” can be removed.

L200-204 – Very long sentence, twice with “which” in it too, needs breaking up.

L204 – I would clarify that you did not need to run a zero-inflated model for the juveniles in the end.

L214 – I have found model validation with glmmTMB to be difficult because I was unable to extract Pearson residuals which are needed for dispersion testing (this was a couple of years back though, might be updated now). How did you get around that issue?

L225 – That table is super useful but unfortunately not picked up again anywhere. That makes it feel a bit redundant. What was the aim in creating it? I would mention it somewhere, it might save a lot of researchers some time if it’s more in the spotlight.

Results:

General – the selection of which sites are presented throughout the text seems arbitrary at times and does not really follow a, for me, recognisable pattern.

Discussion:

General – I am not a fan of the first paragraph of the discussion. The rest of the discussion is great, especially the conclusion and the hypotheses you formulate for your findings! Just the first paragraph is lacking the clarity and punchiness of the rest.

L331-340 – This text should really be in the introduction. If the first discussion paragraph should include any introductory content it should only be one sentence at most.

Then you can get right into the most important result of your study, then move onto the next most important finding, and the next, and so on. Each finding should really only have one or two sentences. I would then structure the rest of the discussion in the same order of your first paragraph, each ensuing paragraph explains one finding, like you have already. I would just rearrange the paragraphs.

L355 – Oxford comma missing (the comma before the ‘and’). Most of your text uses commas before ‘and’ in an enumeration, which is fine, just needs to be consistent throughout the text.

L384 – It would help to mention the location of the Graham et al. study here; I think it would help the reader contextualising.

L409 – You can drop the apostrophes around ‘weedy’ here, you have introduced it in the text before already.

L415 – Oxford comma missing.

L465 – Oxford comma missing

L463-469 – Really long sentence with ‘… reefs, reefs …’ in the middle, makes it tough to read. I would break it up.

If anything is unclear, please do not hesitate to contact me.

Best,

Jan-Claas Dajka
