# Supplementary material for: First insights into coral recruit and juvenile abundances at remote Aldabra Atoll, Seychelles
Source: PLoS One. 2021 Dec 7;16(12):e0260516. doi: 10.1371/journal.pone.0260516 (PMC8651144; doi:10.1371/journal.pone.0260516)
Supplement: Attachment — Submitted filename: Response to Reviewers.docx [file pone.0260516.s013.docx]

Response to reviewers – submission ID: PONE-D-21-19918

**First insights into coral recruitment and juvenile abundances at remote Aldabra Atoll, Seychelles**

Anna Koester, Amanda Ford, Sebastian Ferse, Valentina Migani, Nancy Bunbury, Cheryl Sanchez, Christian Wild

Reponses to reviewers marked in blue. References to lines in the manuscript are made to the ‘Revised Manuscript with Track Changes’ file. In addition to changes in response to the reviewers’ feedback, a reference was added for the indication of the size of Aldabra’s lagoon (reference #35, line 107)

**__________________________________________________________________________________**

**Reviewer 1 comments**

1. The manuscript described the changes in coral juvenile abundance before/after coral bleaching event, and compared between 12 different sites in atoll. Also, authors compared the coral recruitment abundance between lagoon and seaward sites by settlement tile. All of the experiments were conducted at remote Aldabra Atoll where no human disturbance occurs. This manuscript contributes to the more understanding of early life stage dynamics of coral. The manuscript is well written and the results presented here are worthy of publication in PLOS One. I only have few comments for this manuscript. In Discussion, I feel the authors did not provide satisfactory explanation about the decrease of coral juveniles after bleaching event. I can understand the results showed similar patterns between coral coverage and juvenile abundance, however, many factors could cause the decrease of coral juveniles, for example, bleaching also influence the coral reproduction for several years, and the recovery patterns are similar as the results in this study (see Levitan et al. 2014 and Johnston et al. 2020).

**Response:** We thank Reviewer 1 for the constructive feedback that helped to improve and strengthen the manuscript. We now specifically added that the reduction in coral juvenile abundances may also be related to reduced coral reproduction caused by heat stress and bleaching. This was added in **lines 356–362**, **367–368 and 370–371**.

**Reviewer 2 comments**

1. The study of Koester, Ford, Ferse, Migani, Bunbury, Sanchez and Wild assessed coral recruitment in the Aldabra Atoll of the Outer Seychelles by looking at two vital life cycle stages, coral recruits and coral juveniles. Koester et al. give interesting and important insights into the impact of the 2016 mass coral bleaching event on Aldabra Atoll and the recruitment dynamics after it. They also assess more limited datasets before the event. The reported coral recovery of the remote atoll is remarkably rapid and indicates the potential of corals to respond to bleaching events with reduced presence of human stressors. This is an important insight to see published. Koester et al.’s interpretations of the findings are on point; I especially enjoyed their interpretations of their results and the corresponding hypotheses as to why they could be seen. I have minor suggestions for improvements. The manuscript is carefully prepared and well-written. The most important of my improvement suggestions are regarding the structure of introduction and discussion. Some sentences are very long as well, I have marked the ones that definitely need breaking up. Please find specific comments below.

**Response:** We thank Reviewer 2 for the constructive and detailed feedback that helped to improve and strengthen the manuscript.

Introduction:

2. General – Sometimes, ‘recruit’ and ‘recruitment’ get used interchangeably in the text. For instance, in line 200: “As the coral recruitment dataset may have an excess of zeros”. Should this not be the ‘coral recruit dataset’? To my knowledge, ‘coral recruitment’ is the replenishment of the local adult population by new individuals from within or outside an existing population (Hughes et al. 2010). I always understood this as coral juveniles being included in the recruitment process. Therefore, I would be careful when treating the words interchangeably.

Hughes TP, Graham NAJ, Jackson JBC, Mumby PJ, Steneck RS (2010) Rising to the challenge of sustaining coral reef resilience. Trends in Ecology & Evolution 25:633–642

**Response:** Thank you for this very good comment. This was now adapted throughout the manuscript (including the title and Figure 1) and ‘recruitment’ is only used if it includes also the juvenile stage.

3. General – I do not think you need to introduce recruitment dynamic topics like rubble and CCA in the introduction, since neither are picked up in the rest of the manuscript. I would delete the phrases regarding both CCA and rubble.

**Response**: Thank you, this is a good point – we considered not specifically mentioning these aspects and removing this section following your comment. However, despite it not being further addressed in much detail, we think this is still important to mention. The fact that we have observed a substantial increase in coral juveniles following the bleaching event implies the sufficient availability of suitable settlement substrate at Aldabra (and is supported by our previous work: Koester et al. 2020). We do briefly mention this assumption in **lines 410–411** and now specifically added the coverage of CCA as an example to this statement (**line 411**).

Koester A, Migani V, Bunbury N, Ford AK, Sanchez C, Wild C. Early trajectories of benthic coral reef communities following the 2015/16 coral bleaching event at remote Aldabra Atoll, Seychelles. Sci Rep 2020;10:17034. <https://doi.org/10.1038/s41598-020-74077-x>

Methods:

4. L136 – Is the sentence regarding approval necessary in a manuscript? I have not had the need to include it yet.

**Response**: This statement (now **lines 138–139**) was included to adhere to the PLOS ONE requirements which state: “*Methods sections for submissions reporting on any type of field study must include ethics statements that specify: Permits and approvals obtained for the work, including the full name of the authority that approved the study; if none were required, authors should explain why”* (<https://journals.plos.org/plosone/s/submission-guidelines>; under “Guidelines for Specific Study Types” and then “Observational and field studies”)

5. L145 – I think you might need a reference for that fragmented colonies statement.

**Response:** As this is based on personal observation, we removed this statement (**lines 146–147**).

6. L148-153 – Very long sentence, needs breaking up.

**Response**: Addressed (**lines 150–154**).

7. L198 – The comma after “i.e.” can be removed.

**Response**: Removed (**line 200**).

8. L200-204 – Very long sentence, twice with “which” in it too, needs breaking up.

**Response**: Addressed (**lines 202–206**).

9. L204 – I would clarify that you did not need to run a zero-inflated model for the juveniles in the end.

**Response**: Information added (**line 204**).

10. L214 – I have found model validation with glmmTMB to be difficult because I was unable to extract Pearson residuals which are needed for dispersion testing (this was a couple of years back though, might be updated now). How did you get around that issue?

**Response**: We used the DHARMa package to validate the models. Several test functions are available through this package, amongst others also for dispersion testing. These functions also work for models build with the glmmTMB package. More details were added to **lines 221–222** for clarity.

When addressing this comment, we took the opportunity to revisit our models and identified that we have reported the wrong model for the coral recruit analysis. The data is overdispersed but not zero-inflated and the final model does not account for zero-inflation. The results are largely the same, the only change is that the difference in recruit abundance at the lagoon and seaward site in the June-August period is not significant. Accordingly, we have adjusted the methods (**lines 218–219**), the results (**lines 306–308**) and **S4 Table** . However, this change does not impact the main points, assumptions and conclusions brought forward in the discussion.

11. L225 – That table is super useful but unfortunately not picked up again anywhere. That makes it feel a bit redundant. What was the aim in creating it? I would mention it somewhere, it might save a lot of researchers some time if it’s more in the spotlight.

**Response**: The aim of the table is to provide a global comparison in coral juvenile abundance, particular to put our results in perspective. We would have liked to include the table in the main body to make it more visible, but it is of course too big. However, we do utilise this table to compare our results with findings elsewhere and included this in the discussion (**lines 403–408**).

Results:

12. General – the selection of which sites are presented throughout the text seems arbitrary at times and does not really follow a, for me, recognisable pattern.

**Response**: With regards to the coral juvenile dataset, the results section loosely follows this order

1) 2015 vs 2016 (only includes the seaward reefs as there was no data collected in the lagoon in 2015): the western sites are described before the eastern sites and shallow depth before deep depth
2) 2016–2019: seaward sites are generally described before the lagoon (where it made sense with regard to the results), with western sites mentioned before eastern sites, and shallow depth before deep depth

With regards to the coral recruit dataset, the lagoonal site is described before the seaward site.

Discussion:

13. General – I am not a fan of the first paragraph of the discussion. The rest of the discussion is great, especially the conclusion and the hypotheses you formulate for your findings! Just the first paragraph is lacking the clarity and punchiness of the rest.

**Response**: See response to comment #14.

14. L331-340 – This text should really be in the introduction. If the first discussion paragraph should include any introductory content it should only be one sentence at most. Then you can get right into the most important result of your study, then move onto the next most important finding, and the next, and so on. Each finding should really only have one or two sentences. I would then structure the rest of the discussion in the same order of your first paragraph, each ensuing paragraph explains one finding, like you have already. I would just rearrange the paragraphs.

**Response**: Thank you for this suggestion – we have rearranged the first paragraph, removed the redundant text and added further points with reference to the comment of Reviewer 1. As for the remaining paragraphs, we prefer the current structure, as it follows a chronological order of events, i.e. impact of bleaching overall, then on different taxa, then recovery period overall and different taxa and then a discussion on the coral recruit results as additional baseline observations. Within these paragraphs, we can confirm the proposed format (i.e., findings being mentioned in 1-2 sentences, followed by a discussion/interpretation and explanation of these findings).

15. L355 – Oxford comma missing (the comma before the ‘and’). Most of your text uses commas before ‘and’ in an enumeration, which is fine, just needs to be consistent throughout the text.

**Response**: Added (**lines 150–154**).

16. L384 – It would help to mention the location of the Graham et al. study here; I think it would help the reader contextualising.

**Response**: Added (**lines 401–402**).

17. L409 – You can drop the apostrophes around ‘weedy’ here, you have introduced it in the text before already.

**Response**: Removed (**line 427**).

18. L415 – Oxford comma missing.

**Response**: Added (**line 433**).

19. L465 – Oxford comma missing

**Response**: Added (**line 483**).

20. L463-469 – Really long sentence with ‘… reefs, reefs …’ in the middle, makes it tough to read. I would break it up.

**Response**: Addressed (**lines 481–487**).
